# Could ultrasound midwifery training increase antenatal detection of congenital anomalies in Ghana?

**DOI:** 10.1371/journal.pone.0272250

**Published:** 2022-08-01

**Authors:** Alhassan Abdul-Mumin, Lauren N. Rotkis, Solomon Gumanga, Emily E. Fay, Donna M. Denno

**Affiliations:** 1 Pediatrics and Child Health, Tamale Teaching Hospital, Tamale, Ghana; 2 Pediatrics and Child Health, University for Development Studies, School of Medicine and Health Sciences, Tamale, Ghana; 3 Department of Family and Child Nursing, University of Washington School of Nursing, Seattle, WA, United States of America; 4 Global Center for Integrated Health of Women, Adolescents, and Children, University of Washington, Seattle, WA, United States of America; 5 Department of Obstetrics & Gynaecology, Tamale Teaching Hospital, Tamale, Ghana; 6 Department of Obstetrics & Gynecology, University of Washington School of Medicine, Seattle, WA, United States of America; 7 Department of Pediatrics, University of Washington School of Medicine, Seattle, WA, United States of America; 8 Department of Global Health, University of Washington School of Public Health, Seattle, WA, United States of America; UNITED KINGDOM

## Abstract

**Background:**

As part of World Health Organization (WHO) 2016 updated antenatal care (ANC) guidelines routine ultrasonography is recommended, including to detect congenital anomalies. The Ghana Health Service (GHS) developed an in-service midwifery ultrasound training course in 2017, which includes fetal anomaly detection. Training rollout has been very limited. We sought to determine proportions of anomalies among neonates presenting to Tamale Teaching Hospital (TTH) that should be prenatally detectable by course-trained midwives in order to determine training program potential utility.

**Methods:**

We analyzed data from a registry of neonates admitted to TTH with congenital anomaly diagnoses in 2016. We classified ultrasonographic detectability of anomalies at ≤13 and 14–23 weeks gestation, based on GHS course content and literature review. Secondary analysis included 2011–2015 retrospective chart review data.

**Results:**

Eighty-five neonates with congenital anomalies were admitted to TTH in 2016. Seventy-three (86%) mothers received ≥1 ANC visit; 47 (55%) had at least one prenatal ultrasound, but only three (6%) were interpreted as abnormal. Sixteen (19%) and 26 (31%) of the anomalies should be readily detectable by course-trained midwives at ≤13 and 14–23 weeks gestation, respectively. When the 161 anomalies from 2011–2015 were also analyzed, 52 (21%) and 105 (43%) should be readily detectable at ≤13 and 14–23 weeks gestation, respectively. “Optimal conditions” (state-of-the-art equipment by ultrasonography-trained physicians) should readily identify 53 (22%) and 115 (47%) of the anomalies at ≤13 and 14–23 weeks gestation, respectively.

**Conclusion:**

Training Ghanaian midwives could substantially increase second trimester anomaly detection, potentially at proportions nearing highly resourced settings. Our data also highlight the need for refinement of the WHO antenatal ultrasonography recommendation for a scan before 24 weeks gestation for multiple purposes. Gestational dating accuracy requires first trimester scanning while fetal anomaly detection is more accurate during second trimester. Further specification will enhance guideline utility.

## Background

Congenital anomalies are a major cause of morbidity and mortality globally. Prevalence is approximately 20 per 1000 births and account for 11% of neonatal deaths and 4% of deaths among under five-year-olds [1]. Congenital anomalies rank as the 14^th^ and 9^th^ leading cause of disability adjusted life years worldwide and in low-income countries, respectively [2–5]. Approximately 94% of severe anomalies, such as heart defects, neural tube defects and those associated with Down syndrome, occur in low- and middle-income countries (LMIC) [6]. This disproportionate burden is likely related to maternal undernutrition, including micronutrient deficiencies, exposure to infections and toxins, and limited antenatal care coverage and quality [6].

To reduce preventable child mortality and meet the Sustainable Development Goals child health target, focused attention on neonatal conditions, including congenital anomalies, is required [7]. Timely prenatal detection could allow mothers and families to become informed of implications, weigh treatment options, seek termination of pregnancy if desired, and prepare for anticipated treatments and allocation of resources at birth.

The World Health Organization (WHO) updated their antenatal care (ANC) guidelines in 2016, recommending ultrasonography before 24 weeks gestation to estimate gestational age/reduce early labor induction, detect multiple gestations and congenital anomalies, and improve women’s pregnancy experience [8]. Ultrasound is low risk, non-invasive and already standard in ANC in high-income countries.

In Ghana prenatal sonography, especially in rural areas, is often unavailable or only accessible from providers able to assess gestational age and fetal presentation, but without the training or equipment to detect congenital anomalies. In 2017 the Ghana Health Service (GHS) developed an in-service midwifery ultrasound training course, but implementation has been very limited with no immediate plans for scale-up.

Tamale Teaching Hospital (TTH) is the major referral hospital for northern Ghana which is largely rural and has the highest fertility and neonatal and child mortality rates in the country [9, 10]. All newborns delivered at TTH and diagnosed with congenital anomalies are admitted to the neonatal intensive care unit (NICU). Those born with congenital anomalies from the catchment area are also routinely referred to the TTH NICU. A yearlong prospective registry of all neonates admitted to the TTH NICU was conducted in 2016. The aim of this study was to determine the proportion of congenital anomalies among neonates admitted to the TTH NICU that can be expected to be detected by prenatal ultrasound screening by GHS course-trained midwives in order to determine the potential impact of implementation of the training program.

## Methods

All neonates (<28 days) with congenital anomalies were enrolled into a registry at the time of admission to the TTH NICU in 2016. This unit and, by extension the hospital, serves seven of the 16 administrative regions of Ghana, encompassing a population of approximately 7 million people [11]. An estimated 200,000 annual births occur in this catchment area, approximately 22% of births nationally [11]. An abstraction template was used to record maternal and neonatal data, including prenatal ultrasound and maternal characteristics from ANC cards, and delivery history from inpatient notes.

Based on the GHS midwifery training manual [12], a literature review [13–15], and maternal-fetal medicine specialist expert opinion (EEF) we classified anomalies as those that should be “readily detectable”, “potentially detectable” or “not detectable” by midwives trained in the course as well as under “optimal” circumstances (Table 1). The latter was defined as use of state-of-the-art transabdominal ultrasound by physicians trained in prenatal ultrasonography, but excluding advanced techniques (e.g., transvaginal ultrasound, nuchal translucency measurement).

**Table 1:** Ultrasound detectability classification by anomaly type.

Congenital Anomaly (N)	Anticipated Detectability Key				Notes
	1: Anomaly should be readily detectable by ultrasound				
	2: Anomaly should be potentially detectable by ultrasound				
	3: Anomaly not expected to be detectable by ultrasound				
	≤13 Weeks Gestation under “Optimal” Conditions^a^	≤13 Weeks Gestation According to Midwifery Training^b^	Within 14–23 Weeks Gestation under “Optimal” Conditions	Within 14–23 Weeks Gestation According to Midwifery Training	
Cleft Palate	3	3	3	3	Isolated cleft palate is difficult to detect by ultrasound in all scenarios.
Cleft Lip and Palate	3	3	2	2	
Hydrocephalus	2	2	2	2	Hydrocephalus may develop or worsen over time, i.e., if develops later in pregnancy may not be seen earlier in pregnancy.
Encephalocele	2	3	1	2	While not explicitly mentioned in the GHS training manual, scan of the brain is included. However, encephalocele may also be small and difficult to detect early in pregnancy.
Spina Bifida Occulta	3	3	3	3	
Spina Bifida / Meningocele	2	2	1	1	
Microcephaly	3	3	2	2	The disparity between head to body size develops over time and may be mild. Unlikely to be detectable in the first 13 weeks, but possibly detectable, depending on severity, between 14–23 weeks. While not explicitly mentioned in the GHS training manual, head circumference measurement is included.
Anencephaly	1	1	1	1	
Gastroschisis	1	1	1	1	Normal physiological gut herniation early in pregnancy can lead to a false positive if the ultrasound is done prior to 11–13 weeks.
Omphalocele	1	1	1	1	Normal physiological gut herniation early in pregnancy can lead to a false positive if the ultrasound is done prior to 11–13 weeks.
Rectovaginal Fistula	3	3	3	3	
Imperforate Anus	3	3	3	3	
Persistent Omphalomesenteric Duct	3	3	3	3	
Hirschsprung’s disease	3	3	3	3	Difficult to detect by ultrasound until third trimester of pregnancy when dilated bowel may be visualized.
Amniotic Band Syndrome	2	2	2	2	Detection of anomalies associated with amniotic band syndrome depend on the location of the constrictions and if they lead to amputations.
Human Pseudotail	3	3	2	2	While not explicitly mentioned in the GHS training manual, scan of the length of the spine is included, so human pseudotail could be detectable. However this would only be detectable later in pregnancy.
Osteogenesis Imperfecta	2	2	2	2	Detection of osteogenesis imperfecta depends on the severity of the case, which can involve shortening or bowing of long bones or fractures.
Polydactyly	3	3	2	3	Difficult to detect by ultrasound in all scenarios.
Congenital Hip Joint Deformity	3	3	3	3	
Talipes Equinovarus	2	3	2	3	GHS training manual does not include detection of talipes equinovarus, which can be subtle and difficult to detect even under more optional conditions.
Achondroplasia	3	3	2	2	Achondroplasia is not usually detectable until later in the second trimester.
Bladder Exstrophy	2	2	1	2	While not explicitly mentioned in the GHS training manual, scan of the genitourinary tract is included, and severe cases of bladder exstrophy should be recognizable.
Prune Belly	2	2	1	2	While not explicitly mentioned in the GHS training manual, scan of the abdomen and genitourinary tract is included, and severe cases of prune belly should be recognizable.
Urethral agenesis	3	3	2	2	While not explicitly mentioned in the GHS training manual, scan of the genitourinary tract is included, and severe cases of urethral agenesis should be recognizable.
Trisomy 21	3	3	3	3	Trisomy 21 can present with a variety of anomalies that may or may not be detectable on ultrasound; however findings specific to trisomy 21 are not routinely detected by ultrasound. Increased nuchal translucency measurement in early pregnancy is a more sensitive approach to trisomy 21 detection, but requires specialized training.
Cystic Hygroma	1	2	1	2	Detection of cystic hygroma becomes increasingly feasible with greater severity. However, it is not explicitly included in the GHS training manual.
Teratoma	3	3	2	2	Should be detectable but can develop at any time during gestation, hence coded as “2/potentially detectable”. As it tends to develop over time, unlikely to be at detectable <14 weeks.
“Dysmorphic Features” NOS	3	3	3	3	
Conjoined Twins	1	1	1	1	

^a^“Optimal Conditions” were defined as using state-of-the-art ultrasound technology by an ultrasonography-trained physician.

^b^Midwifery training was defined per Vance C., Jeanty P. Limited Obstetric Ultrasound: Course Manual. General Electric Healthcare; 2016 [8].

The 2-week GHS midwifery training is based on use of the General Electric (GE) V-scan ACCESS model to determine fetal number, estimate gestational age, assess for placental conditions, measure amniotic fluid levels, and identify internal and external fetal structures–including to detect anomalies [12]. While not intended to comprehensively identify all anomalies, the training provides explicit instruction on assessment for a number of specific anomalies (e.g., gastroschisis, hydrocephalus, spina bifida) as well as visualization and inspection of specific fetal structures (e.g. lower extremities, brain, genitourinary tract). While some anomalies were not specifically referenced by name in the training manual, examination techniques and anatomical coverage should lead to detection (e.g., scan of the genitourinary tract should reveal bladder exstrophy even though this condition was not specifically named in the manual). Anomalies in such scenarios were coded as “potentially detectable”.

Other circumstances in which the “potentially detectable” code was applied included conditions that progress during gestation (e.g., microcephaly) or present with varying severity (e.g., osteogenesis imperfecta). Details of detectability coding are presented in Table 1.

The ability to detect most anomalies varies by gestational age, hence we reported detectability under the following scenarios: 1) by 13 weeks gestation under “optimal” circumstances, 2) by 13 weeks gestation by course-trained midwives, 3) between 14–23 weeks gestation under “optimal” circumstances, and 4) between 14–23 weeks gestation by course-trained midwives. If a child was diagnosed with more than one condition, we included the most readily detectable anomaly in our classification count.

As a secondary analysis, we included data collected by retrospective chart review and published in 2017 that enumerated neonates admitted to the TTH NICU with congenital anomalies over five years (2011–2015) [16]. We applied the same framework described above to these data.

Descriptive statistics were calculated using Excel (Microsoft Corporation, Bellevue, WA). We also explored whether a priori determined demographic and clinical variables (number of ANC visits (classified as any vs none and ≥4 vs <4), history of prenatal ultrasound (including if at <24 weeks gestation vs ≥24 weeks and if by the first 2 trimesters vs not), and advanced maternal age) were associated with an anomaly amenable to detection by prenatal ultrasonography at <24 weeks gestation. These demographic and clinical data were captured in the 2016 registry, but not available for patients admitted from 2011–2015. We also tested whether there was a difference in potential detectability based on the midwifery training course compared to “optimal conditions”, using chi-square and Fisher exact (if cells contained <five observations) tests in Stata 16.0 (StataCorps, College Station, TX). P-values were two-tailed and alpha defined as 0.05.

This study was approved by the TTH Ethical Review Committee (TTHERC/19/06/18/18) and exempted from University of Washington Human Subjects Division review. As data was abstracted from routine clinical records anonymously without any identifiers, consent was not required.

## Results

In 2016, approximately 7,380 livebirths were delivered at TTH, 18 were diagnosed with congenital anomalies and admitted to the NICU. An additional 67 neonates diagnosed with anomalies were admitted as referrals. Mean maternal age was 27 years (Table 2). Seventy-five (86%) and 49 (58%) of mothers received at least one and four ANC visits, respectively, mostly in public facilities. Forty-four (52%) received one prenatal ultrasound and three (4%) received two. Of the 47 initial scans, one (2%), 32 (68%), and seven (15%) were completed in the first, second, and third trimesters, respectively. Gestational age at ultrasound was unknown for seven (15%). Twenty-seven (58%) initial scans were completed before 24 weeks gestation. Of the 50 scans (47 initial and 3 subsequent), only three (6%) were interpreted as abnormal: two reporting hydrocephalus (completed at 30 and 33 weeks gestational age), but neither the detected condition nor gestational age at scan was specified for the third (osteogenesis imperfecta diagnosed postnatally).

**Table 2:** Descriptive characteristics (N = 85 unless otherwise specified).

Age at Admission in Days (Mean±SD)^*a*^	4.5±5.7	
Sex of Neonate (female)^b^	50%	
Birth Weight in Kg (Mean±SD)^c^	2.7±0.4	
Maternal Age in Years Mean 27.4, SD 4.5	15–20	6 (7.1%)
	21–34	73 (85.9%)
	≥35	6 (7.1%)
Facility Type for Antenatal Care	None	11 (12.9%)
	Primary health center	37 (43.5%)
	District hospital	27 (31.8%)
	Regional or teaching hospital	6 (7.1%)
	Private clinic	4 (4.7%)
ANC Visits	0	11 (12.9%)
	1	1 (1.2%)
	2–4	52 (61.2%)
	5–8	19 (22.4%)
	9+	1 (1.2%)
	Unknown	1 (1.2%)
Number of Ultrasound Scans	0	38 (44.7%)
	1	44 (51.8%)
	2	3 (3.5%)
Gestational Age at First Ultrasound Scan^d^ (N = 47)	1^st^ trimester (≤13 weeks)^e^	1 (2.1%)
	2^nd^ trimester (14–27 weeks)	32 (68.1%)
	3^rd^ trimester (28–40 weeks)	7 (14.9%)
	Unknown	7 (14.9%)
At Least One Ultrasound Scan by 24 Weeks^f^ (N = 47)	<24 weeks	27 (57.5%)
	24+ weeks	13 (27.7%)
	Unknown	7 (14.9%)
Fetal Ultrasound Scan Results (N = 47)	Normal	44 (93.6%)
	Abnormal^g^	3 (6.4%)
Facility Type for Delivery	Home	16 (18.8%)
	Primary health center	14 (16.5%)
	District hospital	33 (38.8%)
	Regional or teaching hospital^h^	20 (23.5%)
	Private facility	2 (2.4%)
Specialty Involved in Neonatal Management (other than Pediatrics)	None	14 (16.5%)
	Neurosurgery	25 (29.4%)
	Orthopedic	6 (7.1%)
	Otolaryngology	1 (1.2%)
	Pediatric surgery	27 (31.8%)
	Dental	7 (8.2%)
	Ophthalmology	1 (1.2%)
	Urology	4 (4.7%)
Disposition	Died during NICU stay	10 (11.8%)
	Referred to TTH surgery ward	16 (18.8%)
	Discharge home	59 (69.4%)
	Without surgical subspecialty follow up	10 (11.8%)
	With non-urgent referral to subspecialty surgery	42(48.4%)
	With subspecialty follow up after initial inpatient surgery	7(8.2%)

**Detectable by 2**^**nd**^ **Trimester (£27 weeks)—according to midwife manual**^a^Among n = 82 for whom this data was available.

^b^Among n = 84 for whom this data was available.

^c^Among n = 69 for whom this data was available.

^d^Of the 3 pregnancies with two scans, the first scans were conducted between 17–23 weeks gestation and the second scans were conducted between 25–31 weeks gestation.

^e^First trimester scan was completed at 11 weeks at TTH.

^f^WHO 2016 Antenatal Care Guidelines recommend a scan before 24 weeks gestation [8].

^g^Of the 3 neonates with prenatal scans that were interpreted as abnormal, 2 were prenatally diagnosed with hydrocephalus and 1 was interpreted as abnormal, not otherwise specified—this child was diagnosed postnatally with osteogenesis imperfecta. These 3 neonates had 1 antenatal scan each.

^h^18 deliveries at TTH.

Abbreviations: ANC, antenatal care; SD, standard deviation.

During their hospitalization, 10 (12%) died neonates, 16 (19%) were transferred to a TTH inpatient surgical unit, and 59 (69%) were discharged home from the TTH NICU, including 49 (83%) with subspecialty follow-up. Anomaly types most commonly diagnosed included: neurological (N = 30, 35%), gastrointestinal (N = 29, 34%), orthopedic (N = 7, 8%), craniofacial (N = 7, 8%), and genitourinary (N = 4, 5%) (Table 3).

**Table 3:** Congenital anomaly types (n = 85).

Congenital Anomaly Type	N	%	Inpatient Specialties Involved	Hospital Disposition (N)
**Craniofacial**	7	8.2%		
Cleft Palate	3	3.5%	Dental	Discharge home with non-urgent referral to subspecialty (3)
Cleft Lip and Palate	4	4.7%	Dental	Discharge home with non-urgent referral to subspecialty (3) or death (1)
**Neurologic**	30	35.3%		
Hydrocephalus	10	11.8%	Neurosurgery	Referral to TTH surgery ward (7) or discharge home with non-urgent referral to subspecialty (3)
Encephalocele	2	2.4%	Neurosurgery	Referral to TTH surgery ward (1) or discharge home with non-urgent referral to subspecialty (1)
Spina Bifida Occulta^a^	1	1.2%	None	Death (1)
Meningocele/Spina Bifida	10	11.8%	Neurosurgery	Discharge home with non-urgent referral to subspecialty (6) or referral to TTH surgery ward (3) or death (1)
Microcephaly^b^	6	7.1%	Neurosurgery (2) or None (4)	Discharge home with non-urgent referral to subspecialty (2) or discharged home (4)
Human Pseudotail^c^	1	1.2%	Neurosurgery	Discharge home with non-urgent referral to subspecialty (1)
**Gastrointestinal**	29	34.1%		
Gastroschisis	4	4.7%	Pediatric Surgery	Death (4)
Omphalocele^d^	12	14.1%	Pediatric Surgery	Discharge home with non-urgent referral to subspecialty (11) or death (1)
Recto-Vaginal Fistula	1	1.2%	Pediatric Surgery	Referral to TTH surgery ward (1)
Imperforate Anus^e^	9	10.6%	Pediatric Surgery	Referral to TTH surgery ward (2) or discharge home with non-urgent referral to subspecialty (1) or discharge home after initial surgery with non-urgent referral to subspecialty (5) or death (1)
Persistent Omphalomesenteric Duct	1	1.2%	Pediatric Surgery	Discharge home with non-urgent referral to subspecialty (1)
Hirschsprung’s disease	2	2.4%	Pediatric Surgery (1) or None (1)	Discharge home with non-urgent referral to subspecialty (2)
**Orthopedic**	7	8.2%		
Amniotic Band	1	1.2%	Orthopedics	Referral to TTH surgery ward (1)
Osteogenesis Imperfecta	1	1.2%	Orthopedics	Discharge home with non-urgent referral to subspecialty (1)
Polydactyly	1	1.2%	General Surgery	Discharge home (1)
Congenital Hip Joint Deformity	1	1.2%	Orthopedics	Discharge home with non-urgent referral to subspecialty (1)
Talipes Equinovarus^f^	1	1.2%	Orthopedics	Discharge home with non-urgent referral to subspecialty (1)
Congenital Hyperextended Lower Limbs	2	2.4%	Orthopedics	Discharge home with non-urgent referral to subspecialty (2)
**Genitourinary**	4	4.7%		
Bladder Exstrophy	2	2.4%	Urology	Discharge home after initial surgery with non-urgent referral to subspecialty (2)
Prune Belly	2	2.4%	Urology	Discharge home with non-urgent referral to subspecialty (2)
**Chromosomal Syndromes**	2	2.4%		
Trisomy 21	2	2.4%	None	Discharge home (2)
**Fetal Tumor**	2	2.4%		
Cystic Hygroma	1	1.2%	Otolaryngology	Discharge home with non-urgent referral to subspecialty (1)
Teratoma	1	1.2%	Ophthalmology	Referral to surgery within TTH (1)
**Other**	4	4.7%		
“Dysmorphic Features” Not Otherwise Specified	4	4.7%	None	Discharge home (3) or death (1)
**Total Anomalies**	85			

^a^The neonate with spina bifida occulta had secondary findings including low set ears and micrognathia.

^b^Five neonates with microcephaly had secondary findings including, (1) talipes equinovarus, (2) congenital hyperextended lower limbs, (3) talipes equinovarus, absence of elbow joints, microphthalmia, (4) microphthalmia, and (5) findings consistent with fetal alcohol syndrome.

^c^The neonate with human pseudotail had secondary findings of ectopic testis.

^d^The neonate who died was also diagnosed with Beckwith-Wiedemann Syndrome.

^e^The neonate with imperforate anus had secondary findings including webbed neck, and other abnormalities, not otherwise specified.

^f^Two additional neonates were diagnosed with talipes equinovarus, but they also had microcephaly which was noted as the primary condition.

Seventeen (20%) and 33 (39%) of the 85 congenital anomalies in 2016 were characterized as readily detectable by 13 weeks and 14–23 weeks gestation, respectively, under “optimal” conditions, while 16 (19%) and 26 (31%) should be readily detectable at those time points by course-trained midwives, respectively (Table 4). An additional 26 (31%) and 31 (36%) congenital anomalies should be potentially detectable by 14–23 weeks gestation under “optimal” conditions and by trained midwives, respectively.

**Table 4:** Ultrasound detectability of anomalies, including data previously published.

	Detectability <13 Weeks Gestation under “Optimal” Conditions^a^			Detectability <13 Weeks Gestation According to Midwifery Training^b^			Detectability Within 14–23 Weeks Gestation under “Optimal” Conditions			Detectability Within 14–23 Weeks Gestation According to Midwifery Training		
	Readily	Potentially	Not likely	Readily	Potentially	Not likely	Readily	Potentially	Not likely	Readily	Potentially	Not likely
2016 (N = 85)	**17 (20%)**	29 (34%)	39 (46%)	**16 (19%)**	27 (32%)	42 (49%)	**33 (39%)**	26 (31%)	26 (31%)	**26 (31%)**	31 (36%)	28 (33%)
2011–2015 (N = 161)	**36 (22%)**	72 (45%)	53 (33%)	**36 (22%)**	60 (37%)	65 (40%)	**82 (51%)**	56 (35%)	23 (14%)	**79 (49%)**	50 (31%)	32 (20%)
2011–2016 (N = 246)	**53 (22%)**	101 (41%)	92 (37%)	**52 (21%)**	87 (35%)	107 (43%)	**115 (47%)**	82 (33%)	49 (20%)	**105 (43%)**	81 (33%)	60 (24%)

RD = Readily Detectable, PD = Potentially Detectable, ND = Not Detectable.

^a^“Optimal Conditions” were defined as using state-of-the-art ultrasound technology by an ultrasonography-trained physician.

^b^Midwifery Training was defined using Vance C, Jeanty P. Limited Obstetric Ultrasound: Course Manual. General Electric Healthcare; 2016 [8].

With the 161 anomalies from 2011–2015, totaling 246 from 2011–2016, 53 (22%) and 52 (21%) should be readily detectable by 13 weeks gestation, under “optimal” circumstances and by trained midwives, respectively. At 14–23 weeks gestation, 115 (47%) and 105 (43%) should be readily detectable under optimal conditions and by trained midwives, respectively. An additional 87 (35%) and 81 (33%) should be potentially detectable by 13 and 14–23 weeks gestation by trained midwives, respectively.

Levels of anomaly detectability (readily, potentially or not) by trained midwives at 14–23 weeks were not statistically significantly associated with maternal or pregnancy characteristics, including maternal age of ≥35 compared to <35 years, ≥1 ANC visit compared to none, ≥4 ANC visits compared to <4, and timing of scans among those with prenatal sonograms. There were no statistically significant (p<0.05) differences in detectability between optimal conditions compared to what would be expected by course-trained midwives.

## Discussion

Combining the 2011–2015 chart review and 2016 registry data, we estimate that 47% and 43% of neonates with congenital anomalies presenting to TTH NICU should have anomalies that can be readily detected between 14–23 weeks gestation, under “optimal” conditions and by GHS course-trained midwives, respectively. These are likely underestimates, as we classified detectability conservatively. We estimate that midwives could potentially prenatally identify another one-third of the anomalies. This represents a notable potential, especially compared to the 4% of scans that were interpreted as abnormal for fetal anomalies among the 47 neonates admitted for congenital anomalies in 2016 who had a prenatal scan.

Improved antenatal identification of congenital anomalies would provide Ghanaian mothers and families opportunities to learn about and prepare for implications of detected conditions. A prenatal diagnosis can allow families to develop birth plans that include place of delivery, financial planning, and transportation arrangements for specialized care referrals. Additionally, families could consider termination when appropriate, especially for conditions that are more definitively diagnosed by prenatal ultrasound (e.g., anencephaly), as induced abortion is legal in Ghana through the second trimester, including if “there is substantial risk that the child, if born, may suffer from or later develop a serious physical abnormality or disease” [17].

Published congenital anomaly antenatal detection prevalence from LMICs are limited; estimates from high-resource setting studies range between 11–85% [14, 18–22]. This variability reflects factors including: sonographer expertise, equipment type, healthcare facility level, anomaly type and gestational age at scan, assessment among all pregnancy outcomes or restricted to livebirths, study design, and use of assessments in addition to transabdominal ultrasound. Indeed, most publications reported determinations based on transabdominal ultrasound plus transvaginal ultrasound, nuchal translucency assessment, or maternal serum markers [14, 15, 18, 19, 21]—technologies not currently feasible in most LMICs, including Ghana. A 2015 Cochrane review found that routine transabdominal ultrasound before 24 weeks gestation was 3.5 more likely to identify anomalies compared to selective screening due to indications, although detection of perinatal anomalies was low in both groups (16% vs. 4%) [21]. These findings were based on two high resource-setting trials, but from 1979–1980 and 1987–1991 when equipment was less advanced and experience with the technology was more limited [22]. A 2006–2013 Swedish study of >10,000 pregnancies, calculated 30.5% detection with universal transabdominal ultrasound screening before 22 weeks gestation [20].

Our estimates of the proportion of congenital anomalies that should be detectable based on the GHS midwifery training course are well within those reported in the literature, suggesting that scaled implementation of the Ghana course could readily improve antenatal detection of congenital anomalies and even be comparable to high-resource settings and could be a model for other LMICs. Universal sonography has been commonplace in high-resource settings for some time and is now recommended by WHO globally as well, although WHO acknowledges that the evidence to support this recommendation is of weak quality [8].

A randomized trial in multiple LMICs published after the 2016 WHO ANC guideline update, found that ultrasonography at 16–22 and 32–36 weeks gestation performed by healthcare professionals who underwent a two-week intensive obstetric ultrasound training program was not associated with stillbirth or neonatal or maternal mortality reductions, or increases in ANC visits or referrals for hospital delivery due to complicated pregnancies [23]. The authors questioned the benefit of prenatal ultrasound in the context of limited resources. However, the two-year timeframe may have been insufficient to increase awareness of ultrasound as a resource to improve detection and management of congenital anomalies, including appropriate referral pathways. More importantly, the study did not assess prenatal ultrasound impact on neonatal morbidity, maternal and family psychosocial and financial preparedness, nor cost implications, in the context of congenital anomaly diagnoses. On the other hand, policy makers will also need to consider resource diversion for referrals, and maternal and family anxiety, due to false positive screens. The aforementioned Swedish study found 5.3% positives were inaccurate [20].

WHO ultrasound guidelines are nonspecific—one scan at <24 weeks gestation is recommended for a number of purposes: intrauterine vs. ectopic location, gestational age estimation, fetal number determination, placental and fetal anomalies detection, and improving women’s ANC experience [8]. However, optimal timing varies depending on the principal goal of the scan, an important aspect not covered in the guideline. For example, first trimester ultrasonography is more accurate for determining ectopic pregnancy, gestational age, and multiple gestations, and mid-second trimester is best for fetal anatomic and placental location and abnormality evaluations [24, 25]. Based on the GHS training course content, 43% of neonates with anomalies admitted to TTH should have anomalies identifiable by trained midwives between 14–23 weeks gestation compared to only 21% before 14 weeks. Furthermore, training and expertise requirements differ depending on sonography type; for example, scanning for congenital anomalies requires more intensive training than for gestational age.

Improved specification of WHO guidance on sonography timing would enable individual countries to determine how to best adapt these recommendations to align with their priorities, specific maternal and neonatal disease burden, and resources for maternal and newborn care. For instance, if resources only allowed for gestational age estimation, national policy might specify a single scan at first ANC presentation and limit training, supervision, and monitoring for that assessment. Middle-income settings with more developed health care systems could recommend an additional later scan to increase placental and fetal anomaly detectability.

In Ghana, the package of maternal health services provided free of charge includes two ultrasound scans [26]. However, sonographers and physicians have been the only personnel trained for provision of prenatal sonography, and primarily for the purposes of gestational dating. Even this service is often unavailable, especially at peripheral facilities, due to lack of trained personnel and equipment [27]. The GHS midwifery training covers first trimester scans to estimate dates and detect ectopic and multiple pregnancies, and second/third trimester scans to include fetal presentation, cervical competence, and placental and fetal anatomic surveys [12]. Thus far the training has only reached a small fraction of midwives. Programmatic rollout to permit task shifting to midwives has the potential to remove access barriers to this recommended intervention.

Expanding prenatal ultrasonography coverage relies on ANC utilization and timing of presentation for care. In our 2016 dataset, 87% and 58% of women had at least one and four ANC visits, compared to 98% and 89% nationally in 2017, respectively [9]. Fifty-one percent and 5% of study mothers received one and two prenatal ultrasounds, respectively, with 63% of first scans occurring by 24 weeks gestation although congenital anomaly detection was only 4%. This low detectability is likely due to the scans being conducted for gestational dating purposes and not with the intent of screening for anomalies. Other factors influencing this detectability prevalence could include sonographers’ training and experience, equipment type, facility level, and gestational age [14, 15, 17, 19]. While continued efforts to improve ANC coverage will be important, even with current levels of ANC coverage, a midwifery antenatal ultrasonography program—that includes scale-up of the training course, supervision, equipment distribution scale-up and maintenance, monitoring and evaluation, and quality assurance—could have substantial impacts on detection, management and outcomes related to congenital anomalies. Efforts to promote early ANC will be needed to leverage the utility of sonography for dating and prevention of early labor induction—only 45% of first ANC visits in Ghana are within the first trimester [9].

Our study has a number of limitations. Data precede any substantial rollout of the midwifery ultrasonography course and, therefore, our congenital anomaly detectability estimates are theoretical, based on the training manual and literature review. We are not aware of evaluations among the small proportion of midwives that received training in 2017, nor any specific plans for further training rollout, which precludes assessments, at this time, of actual detectability that could be achieved. Our data only included anomalies among livebirths and therefore our calculations likely underestimate prenatal detection potential among all pregnancies. The detectability classification assumed adequate visualization of the organ or body system of interest and did not account for the influence of maternal habitus (in particular obesity), fetal position, or factors such as uterine fibroids on sonography accuracy. However, we did account for differences in detectability by gestational age. Our estimates are based on screening ultrasonography only and do not take into account genetic testing which is necessary for definitive diagnosis of certain conditions (e.g., trisomy 21) and is unavailable in Ghana at this time. Our predictions do not consider real world constraints (e.g., waning skill retention, missed opportunities due to equipment malfunction, health worker time constraints, maternal refusal) and are based on an assumption that the midwifery training is effective in skill building, and implementation would be integrated with periodic retraining and supportive supervision to maintain skills, equipment maintenance, and a referral system. Inattention to these factors is common in health programs, including ultrasound initiatives, and can undermine effectiveness [28, 29]. Furthermore, we were unable to assess implications and outcomes of prenatal diagnosis. Finally, the sample size over the one-year prospective period was small and hospital-based and therefore the anomaly profile may not be generalizable to the broader population. However, anomaly types were consistent with similar studies from other settings or of longer duration, including the TTH retrospective 5-year study [4, 14, 16, 18–21].

## Conclusions

Despite these limitations, our data corroborate those from other settings, support further rollout of the GHS midwifery training course coupled with careful evaluation [8], and provide a baseline that can be used for comparison post-course implementation. We also demonstrate the need for refinement of WHO recommendations on the timing of antenatal ultrasonography from the generic guidance of one scan by 24 weeks gestation to clarified guidance based on intended purpose of the scan–for example early scans for dating versus second trimester scans for placental and fetal anomaly detection. Evaluation of any further GHS midwifery training course implementation should include not only determination of actual anomaly detection rates, but also impact on neonatal outcomes, maternal and family perceptions of prenatal anomaly diagnosis benefits, and potential adverse consequences. Assessment of skill retention over time and whether the addition of ultrasonography results in replacement of other ANC interventions (e.g., in settings with shortages of skilled health personnel to deliver ANC) is also essential. Lastly, a cost-benefit assessment should be included, taking into account equipment procurement and servicing in addition to the training, re-training, and supervision necessary to build and maintain midwives’ capacity for antenatal ultrasonography.

## Supporting information

S1 Text (TXT)

## Abbreviations

ANC: Antenatal Care
GE: General Electric
GHS: Ghana Health Service
LMIC: Low-and Middle-Income Countries
NICU: Neonatal Intensive Care Unit
TTH: Tamale Teaching Hospital
WHO: World Health Organization
